# The effects of fermented pineapple residue on growth performance, meat quality, and rumen microbiota of fattening Simmental bull

**DOI:** 10.3389/fmicb.2022.942208

**Published:** 2022-09-14

**Authors:** Ming Deng, Zupeng Xiao, Guangbin Liu, Baoli Sun, Yongqing Guo, Xian Zou, Dewu Liu, Zhenwei Yang, Yaokun Li

**Affiliations:** ^1^Herbivore Laboratory, College of Animal Science, South China Agricultural University, Guangzhou, China; ^2^National Joint Engineering Research Center, South China Agricultural University, Guangzhou, China; ^3^Guangdong Key Laboratory of Agricultural Animal Genomics and Molecular Breeding, South China Agricultural University, Guangzhou, China; ^4^State Key Laboratory of Livestock and Poultry Breeding, Guangdong Key Laboratory of Animal Breeding and Nutrition, Institute of Animal Science, Guangdong Academy of Agricultural Sciences, Guangzhou, China

**Keywords:** fermentation, pineapple, serum indexes, meat quality, rumen microbiota, Simmental bull

## Abstract

In this study, silage *Pennisetum sinese* Roxb-based diet was replaced with fermented pineapple residue (FPR) at the replacement ratio of 0% (CON), 25% (T25), and 50% (T50) in fattening Simmental bulls for 30 days to evaluate the effects of FPR on growth performance, serum indexes, and ruminal characteristics. A total of 30 Simmental bulls (546 ± 44 kg initial BW) were allocated to three groups according to a completely randomized design. On day 30, the slaughter performance and meat quality were determined. Rumen fluids were collected for analyzing the rumen fermentation parameters and microbiota composition on day 30. The results showed that the average daily weight gain increased (*P* < 0.05) as the proportion of FPR rose. Within treatments, the T25 group reached more profit (5.34 RMB per day per bull) than CON while T50 was 3.69. The content of crude fat, cysteine, and proline in the muscle of T50 increased significantly (*P* < 0.05). The amounts of tyrosine, proline, and phenylalanine were significantly increased in the T25 (*P* < 0.05). The beta diversity analysis showed significant differences among the rumen bacterial flora of each group (*P* < 0.05). In the T25 group, the relative abundance of *Spirochaetes* decreased significantly (*P* < 0.05). The relative abundance of *Lachnospiraceae_bacterium_RM44* was significantly lower (*P* < 0.05). Thus, FPR could improve the growth performance, economic benefits, and meat quality without adverse effects on ruminal characteristics.

## Background

After the United Nations Conference on Environment and Development in 1992 (Thomas, 1992), sustainable development became the consensus of countries worldwide. However, many countries are facing issues with the development of sustainable agriculture. Pineapple, the third most produced tropical fruit in China, plays an important role in the agricultural economy. In 2019, pineapple production in Guangdong Province exceeded one million tons and accounted for more than 60% of the production in China (Statistics Bureau of Guangdong Province, 2019). More than 30% of the pineapple residue is inedible pomace (Ketnawa et al., 2012), which may cause environmental pollution and ecological problems if not used properly. A previous study has shown that inedible pineapple pomace has ~19.8% cellulose, 11.7% hemicellulose, and abundant nutrients such as minerals and vitamins (Bardiya et al., 1996). Another study has shown that pineapple waste is physically and chemically suitable for making nursery pots (Jirapornvaree et al., 2017). Pineapple waste material has been used as a substrate for bromelain, organic acids, and ethanol; it can also be used in industrial processes such as fermentation and bioactive component extraction (Atul et al., 2013). Choi et al. (2021) found that feed-finishing Hanwoo steers with pineapple by-products had no adverse effects on growth and carcass performances. Fermentation can be used to process and convert pineapple residue into animal feed (Gowda et al., 2015). A previous study has shown that the addition of 20% fermented pineapple residue (FPR) replacing yellow corn in the basic diet can decrease the abdominal fat percentage of broiler chickens (Mandey et al., 2018). In sheep, pineapple by-product silage in diets could completely replace elephant grass and might reduce production costs without changing the consumption and performance (Cutrim et al., 2013). Wittayakun et al. (2019) found that pineapple waste silage-based diets had no significant impact on rumen fermentation, blood metabolites, and thyroid hormone responses. Hattakum et al. (2019) also found that ruminal pH, ammonia-nitrogen, and volatile fatty acid concentrations were not significantly different when pineapple stem by-products were used to feed Holstein steer. When 40% silage pineapple stem starch was used as roughage to feed Holstein steers, it can improve the feed conversion ratio by promoting short-chain fatty acids production in the rumen (Khongpradit et al., 2020). The addition of 25% silage pineapple residue as roughage can also positively promote weight gain of growing local Myanmar cattle (Kyawt et al., 2020). Considering that the availability of FPR could gain economic and environmental benefits, it might contribute to the sustainable development of agriculture when using FPR as a feedstuff to feed bulls. Therefore, this study aims to analyze the appropriate proportion of FPR replacing silage *Pennisetum sinese* Roxb (SPR), which is widely used in China as roughage in the basic diet of Simmental bull, and to evaluate its impact on growth performance, meat quality, and ruminal characteristics.

## Materials and methods

All experimental procedures and sample collection methods complied with the Regulation on the Administration of Laboratory Animals (CLI.2.293192, 2017 Revision, State Council, China) and were performed in strict accordance with the Institutional Animal Care and Use Committees of South China Agricultural University (approval no. 2018-P002).

### Preparation of FPR and fermentation

The FPR was obtained from BOYA Biotechnology Co., Ltd (Leizhou, Guangdong, China). The raw pineapple peel was squeezed to maintain 78%−80% initial moisture, then evenly sprayed with a mixed lactic acid bacterium (*Lactobacillus plantarum GIM1.191*) and yeast (*Saccharomyces cerevisiae GIM2.133*) liquid. Finally, the FPR mixture was pressed into polyethylene bags (50 kg each) and fermented anaerobically for 20 days.

### Animals, experimental design, and treatments

The experiment was conducted in a beef cattle company in Yunfu, Guangdong, China. A total of 30 healthy Simmental bulls (20 months old, 546 ± 44 kg weight) were used in a completely randomized design for a 3-day adaptation period and a 30-day experimental period. They were randomly divided into three groups in an open sawdust-bedded cowshed: the CON group (no FPR or control, fed basic diet), T25 (25% FPR replaced SPR), and T50 (50% FPR replaced SPR). All bulls were fed a total mixed ration (TMR) at 10:00 and 16:00, and water was provided *ad libitum*. To meet nutritional requirements, the TMR was based on SPR and rice straw as the main forage components and corn flour as the major concentrate component, according to NRC standards (NRC, 2016). The ingredients and nutrient composition of the three diets are shown in Table 1. The remaining feed was collected and recorded daily at 8:30.

**Table 1:** Composition of the raw materials and nutrient content of the diet (%, DM).

**Item**	**CON**	**Treatment**	
		**T25**	**T50**
**Diet composition**			
SPR	39.01	29.04	19.19
FPR	0	10.93	21.68
Straw	16.76	16.63	16.50
Corn flour	24.41	23.81	23.29
Soybean meal	2.10	2.34	2.59
Wheat bran	1.36	1.35	1.34
Rice bran	1.62	1.25	0.88
Wheat middlings	5.36	5.32	5.27
Extruded soybean	0.89	0.89	0.88
Rice flour	1.36	1.35	1.34
Corn gluten	4.46	4.43	4.39
Sodium bicarbonate	1.19	1.18	1.17
Stone powder	0.47	0.47	0.47
Calcium hydrogen phosphate	0.08	0.08	0.08
Fattening bull premix^a^	0.93	0.93	0.93
**Nutrient content**			
Dry matter^b^, kg	10.92	11.00	11.09
Net energy for maintenance^c^, Mcal/kg	1.42	1.45	1.48
Net energy for gain^c^, Mcal/kg	0.83	0.86	0.89
Crude protein^b^	9.76	9.75	9.74
Crude fat^b^	2.91	2.89	2.87
Crude ash content^b^	7.87	7.65	7.43
Neutral detergent fiber^b^	48.67	47.99	47.27
Acid detergent fiber^b^	27.22	26.29	25.36
Starch^c^	19.73	19.55	19.42
Ca^c^	0.94	0.86	0.78
P^c^	0.36	0.36	0.37

^a^Every kilogram of the premix (based on DM) contains copper 191 mg, iron 1,200 mg, manganese 1,393 mg, selenium 9 mg, vitamin A 250 KIU, vitamin E 1,500 IU, vitamin B1 699 mg, niacin 1,500 mg. ^b^The basic nutrient content was calculated from the measured data of each feed ingredient. ^c^Energy, starch, calcium, and phosphorus contents are database comparison values after near-infrared scanning.

### Nutritive value analysis

The samples of FPR and FPR (days 0 and 20) were analyzed for dry matter (DM), crude protein (CP), ether extract (EE), and ash according to the AOAC International guidelines (AOAC, 1990). The neutral detergent fiber (NDF) and acid detergent fiber (ADF) contents were determined using the method reported by Van Soest et al. (1991). These contents were determined from water extract, while wet FPR (20 g) was transferred to a glass bottle filled with 180 ml of deionized water, sealed, mixed, and stored at 4°C overnight (Fang et al., 2016). Then, the water extract was passed through filter paper, and the filtrate pH was measured using a glass-electrode pH meter (Horiba D-21, Horiba, Tokyo, Japan). The FPR had low DM, CP, NDP, ADF, and ash of 21.15, 6.66, 63.46, 33.03, and 4.33% of DM basis, respectively (Table 2). Additionally, it had high starch of 3.2%.

**Table 2:** Nutritional compositions of FPR and SPR (%, DM basis).

**Roughage**	**DM**	**CP**	**EE**	**NDF**	**ADF**	**ASH**	**Starch**
FPR	21.15	6.66	1.78	63.46	33.03	4.33	3.20
SPR	32.76	7.19	1.80	72.70	43.16	6.16	1.00

### Measurements and samples

On days 1, 12, and 24, the feed offered to the bulls was sampled and used for nutrient analysis and chemical analysis. The methods of nutrient determination, including CP, NDF, and ADF, were consistent with the method described in the “Nutritional compositions” section. Chemical analysis of the calcium (Ca) and phosphorus (P) contents was performed using inductively coupled plasma spectroscopy (Chemists and Horwitz, 1990).

The cattle were weighed before the morning feeding on days 1 and 30, and the average daily gain and feed weight ratio were calculated. Eight bulls were randomly selected from each group for the slaughter test. They were fasted for 12 h, and water was withheld for 3 h before slaughter. After being stunned, the cattle were slaughtered according to a general process, including hanging upside down, slaughtering, bloodletting, skinning, removing head and tail, and eviscerating. The live weight was recorded before slaughter and carcass weight after slaughter. Then the samples of the *longissimus thoracis* (LT) were excised between the 12th and 13th rib. After measuring the eye muscle area, pH, and flesh color of LT, cut into two uniform pieces vertically. One piece was put on ice for 24 h to measure drip loss, centrifugal water loss rate, pH and shear force, and the other was frozen in dry ice for the later determination of nutritional indicators.

### Determination of meat quality

The outline of the LT cross-section was delineated with sulfuric acid paper, and the eye muscle area was calculated with ADOBE PDF (version 1.2, San Jose Co., Ltd., CA, United States) after scanning. At 45 min, 24 h, and 48 h after slaughter, the pH was determined using a pH meter (FE28-Standard, METTLER-TOLEDO Co., Ltd., Shanghai, China) in the cut surface of the LT. A colorimeter (NR10QC, 3nh Co., Ltd., Shenzhen, China) was used to measure meat color on the surface of the LT about 45 min after slaughter. The meat samples were cut into 1 cm thick pieces, wrapped in plastic bags, heated to 70°C, and taken out to cool. The meat was cut into strips with a cross-section of 1 cm × 1 cm along the fiber direction, and then the shear force was measured using a tenderness meter (TA. XTPlus, SANHAO Co., Ltd., Suzhou, China). The meat was cut into long strips (5 cm × 2 cm × 2 cm) and weighted as N1. After being packed into plastic bags and hanging suspended for 24 h in a 4°C freezer, the weight was scored as N2. Drip loss% = (N2/N1) × 100%. Then, after centrifugation (1,500 × *g*, 30 min at 4°C), the centrifugal water loss rate was calculated. Analyzing DM, CP, EE, and ash of meat according to the AOAC method and amino acids using a full-automatic amino acid analyzer (LA8080, Hitachi Co., Ltd., Tokyo, Japan).

### Rumen fermentation parameters

Rumen fluid samples were collected from all the bulls on the last day by a rumen tube before the morning feeding. To avoid the contamination of oral saliva, the first 20 ml of rumen fluid was discarded. Approximately 150 ml of rumen fluid sample from each bull was collected and then strained through four layers of cheesecloth. The filtrate was dispensed into 50-ml centrifuge tubes (REF430829, Corning Life Science Co., Ltd.) and 2-ml storage tubes. The samples in the storage tubes were put into liquid nitrogen and then transferred to a −80°C laboratory refrigerator for future use.

The pH of rumen fluid in centrifuge tubes was immediately measured by a pH meter (FE28-Standard, METTLER-TOLEDO Co., Ltd., Shanghai, China). Then, the samples were centrifuged at 5,000 × *g* for 15 min (BR4I, Thermo Co., Ltd., NY, United States) to collect the supernatant. The supernatant was divided into three 15-ml centrifuge tubes. Two tubes were used to measure volatile fatty acid (VFA) (acetic acid, propionic acid, isobutyric acid, butyric acid, isovaleric acid, and valeric acid) content and ammonia nitrogen (NH_3_-N) concentration using a gas chromatograph (SP-3420, BEIFENGRUILI Co., Ltd., Beijing, China) and ELIASA (ST-360, KEHUA Co., Ltd., Shenzhen, China), respectively, according to Erwin et al. (1961) and Broderick and Kang (1980), and were stored at −20°C, and the remaining tube was stored at −80°C as a spare.

### 16S RRNA gene sequencing and annotation analysis

The total genomic DNA was extracted from rumen fluid samples using the modified cetyltrimethylammonium bromide/sodium dodecyl sulfate method (Zhang et al., 2021). The DNA samples were tested for integrity using 1% agarose gel electrophoresis, and their concentration was determined using a Qubit fluorometer (Nanodrop2000/2000C, Thermo Co., Ltd., NY, United States). Then, DNA was diluted to 1 ng/μl using sterile water according to the concentration. The V1–V9 regions of the 16S ribosomal DNA (rDNA) genes were amplified by polymerase chain reaction using the TransStart® FastPfu DNA Polymerase Kit (TransGen Biotech Co., Ltd., Beijing, China). In detail, the amplification was performed with the universal primers (forward primer, 27F: AGAGTTTGATCCTGGCTCAG; reverse primer, 149R: GNTACCTTGTTACGACTT). Sequencing libraries were generated using the SMRTbellTM Template Preparation Kit (Pacific Bioscience, CA, United States) on the PacBio Sequel sequencer.

Single-end reads were assigned to samples based on their unique barcode in the adaptor sequence. Quality filtering of the raw reads was performed to obtain high-quality clean reads according to the PacBio SMRT Portal Provisioning Agreement. The reads were compared with the reference database using the UCHIME algorithm (http//www.drive5.com/usearch/manual/uchime_algo.html) to detect chimeric sequences (Haas et al., 2011; Quast et al., 2012), and clean reads were finally obtained using the Uparse software (Uparse v7.0.1001) Edgar RCUPARSE, 2013. Sequences with ≥97% similarity were assigned to the same operational taxonomic units (OTUs). For each representative OTU, the Silva Database (https://www.arb-silva.de/) was used to annotate taxonomic information based on the Mothur algorithm (Quast et al., 2012). Alpha diversity was applied to analyze the complexity of species diversity within groups, including the observed species, Chao1, Shannon, and ACE indices. Beta diversity analysis was used to evaluate differences between groups using nonmetric multidimensional scaling (NMDS). All these indices were calculated using the quantitative insights into microbial ecology (QIIME) pipeline (Version 1.7.0).

### Statistical analysis

The data were analyzed using the INFLUENCE Statement and GLM Model of SAS (version 9.4; SAS Institute Inc., Cary, NC, United States). A CONTRAST Statement was used to analyze the effects of each index between treatment and control. The test results were presented as the mean and standard error of the mean (SEM), with *P* < 0.05, indicating a significant difference, and *P* < 0.01, indicating a highly significant difference. Growth performance, meat quality indicators, rumen fermentation parameters, and relative abundance of rumen flora were analyzed using the analytical model I: *Yi* = μ + *Pi* + ε*i*, where *Yi* is the dependent variable value of the bull in different treatments, μ is the overall mean, *Pi* is the dietary treatment effect, and ε*i* is the random error. The KENWARDROGERS method is used to perform DOF correction.

## Results

### Nutritional compositions

The FPR had low DM, CP, NDP, ADF, and ash of 21.15, 6.66, 63.46, 33.03, and 4.33% of DM basis, respectively (Table 2). Additionally, it had high starch of 3.2.%.

### Production performance and economic benefits

**Figure 1:**
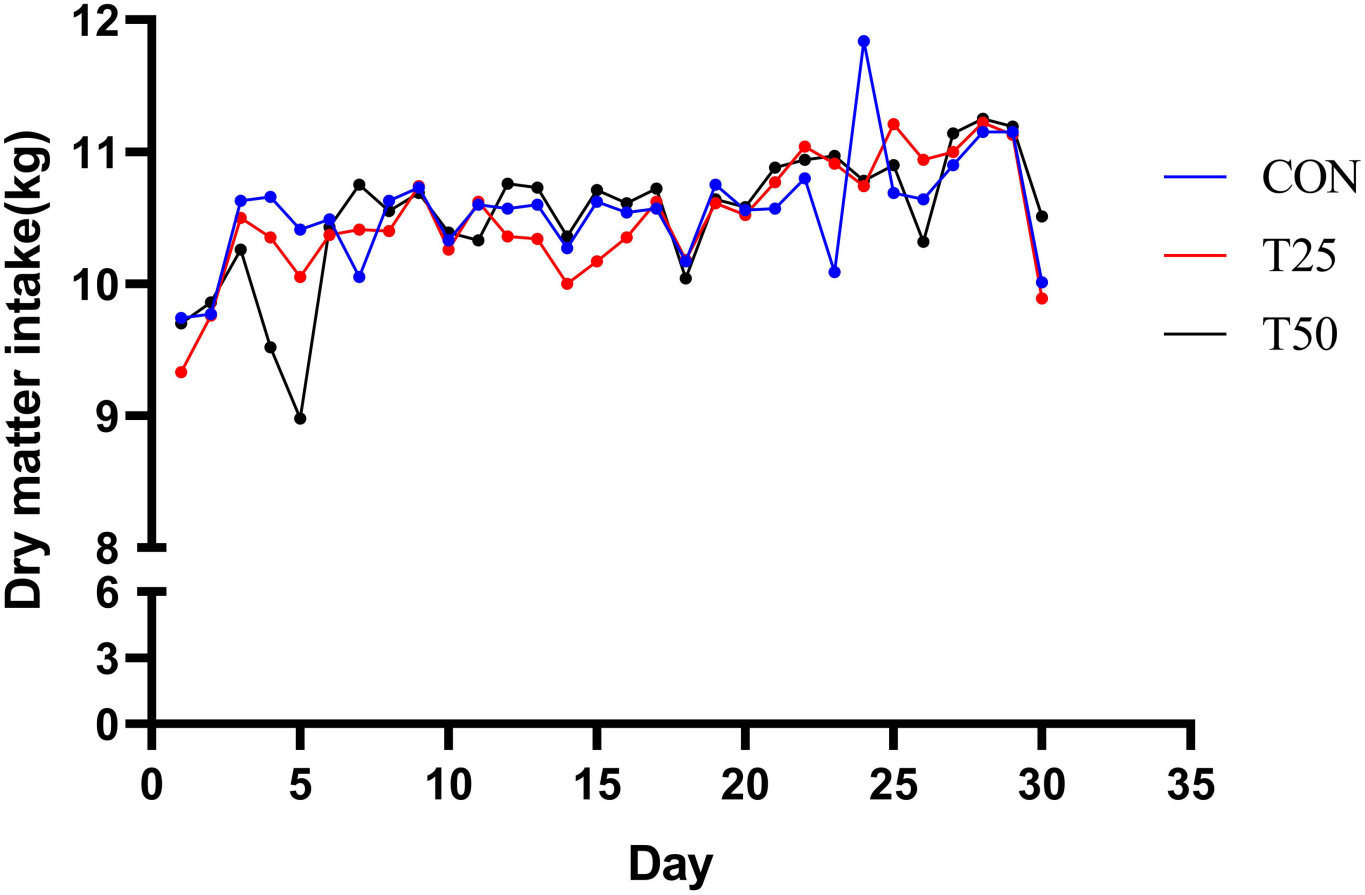
Trends of daily DMI.

The daily dry matter intake (DMI) of the control and treatments were approximately similar (Figure 1). The average daily weight gain of the T25 and T50 groups, respectively, increased by 0.17 and 0.29 kg, and DMI/weight gain significantly (*P* < 0.05) decreased (Table 3). According to the purchase and sale prices, the benefit of fattening each bull per day improved from ¥3.52 (CON) to ¥ 8.86 (T25) and ¥7.21 (T50; Table 4).

**Table 3:** Effects of different proportions of FPR on cattle performance.

**Project**	**CON**	**Treatment**		**SEM**	***P*-value**	**Contrast *P* CON vs. T**
		**T25**	**T50**			
Number	10	10	10			
DMI, kg/day	10.55	10.49	10.52	0.05	0.884	0.65
Initial weight, kg	548.39	539.65	558.5	8.72	0.697	0.972
Final weight, kg	569.44	565.7	588.25	8.57	0.551	0.688
Average daily weight gain, kg	0.70	0.87	0.99	0.06	0.151	0.073
DMI/weight-gain	15.07^A^	12.06^B^	10.62^C^	0.20	< 0.001	< 0.001

Peer data with different acronyms indicate significant differences (P < 0.05), with different acronyms indicating highly significant differences (P < 0.01) and the same acronym indicating insignificant differences. SEM is the pooled standard error between groups; the P-value indicates significance, and the contrast P-value represents the significance between the control and treatment.

**Table 4:** Economic benefits of different proportions of FPR.

**Project**	**CON**	**Treatment**	
		**T25**	**T50**
Feed cost, ¥/kg DM	1.76	2.01	2.27
Feeding costs, ¥/day/cattle	19.17	22.16	25.17
30 days Feeding costs, ¥/cattle	575.02	664.89	755.12
30 days Weight gain, kg/cattle	21.05	26.05	29.75
Cost of weight gain, ¥/kg	30.67	25.52	27.41
Cattle sale price, ¥/kg	35.70	35.70	35.70
30 day sale profit, ¥/cattle	105.88	265.19	246.63
Net profit, ¥/day/cattle	3.52	8.86	7.21

Peer data with different acronyms indicate significant differences (P < 0.05), with different acronyms indicating highly significant differences (P < 0.01) and the same acronym indicating insignificant differences. SEM is the pooled standard error between groups; the P-value indicates significance, and the contrast P-value represents the significance between the control and treatment.

### Meat quality and slaughter performance

Fermented pineapple residue did not adversely affect the slaughter performance and beef sensory quality (Table 5). The crude fat indicators were significantly higher (*P* < 0.05) in T50 than in CON (Table 6). The content of cysteine, glycine, histidine, phenylalanine, proline, and tyrosine in treatments was raised (*P* < 0.05; Table 7), indicating that FPR can improve the amino acid composition of meat.

**Table 5:** Effects of different proportions of FPR on slaughter performance and meat quality.

**Project**	**CON**	**Treatment**		**SEM**	***P*-value**	**Contrast *P* CON vs. T**
		**T25**	**T50**			
Carcass weight, kg	368.44	369.72	378.86	6.78	0.881	0.749
Slaughter rate, %	64.70	65.36	64.06	0.37	0.510	0.338
pH_45min_	7.42	7.37	7.37	0.06	0.946	0.746
pH_24h_	5.88	5.88	5.87	0.02	0.982	0.904
pH_48h_	5.92	5.94	5.90	0.03	0.890	0.973
Eye muscle area, cm^2^	103.86	106.81	103.89	6.65	0.980	0.928
Shear force, N	183.02	169.8	181.58	5.48	0.574	0.558
Centrifugal water loss rate, %	5.05	5.47	5.47	0.004	0.872	0.609
Drip loss rate, %	10.21	10.58	12.24	0.005	0.169	0.214
L*	32.97	31.55	31.81	0.37	0.254	0.113
a*	14.00	13.58	13.77	0.19	0.685	0.456
b*	4.50	3.86	4.34	0.17	0.294	0.295

Peer data with different acronyms indicate significant differences (P < 0.05), with different acronyms indicating highly significant differences (P < 0.01) and the same acronym indicating insignificant differences. SEM is the pooled standard error between groups; the P-value indicates significance, and the contrast P-value represents the significance between the control and treatment.

**Table 6:** Effects of FPR on the main nutrients of Simmental bull.

**Project**	**CON**	**Treatment**		**SEM**	***P*-value**	**Contrast *P***
						**CON vs. T**
		**T25**	**T50**			
Water content, %	72.73	73.51	72.81	0.51	0.374	0.212
Crude protein, % DM	88.99	88.08	85.33	1.17	0.528	0.426
Crude fat, % DM	6.47^b^	7.82^ab^	8.97^a^	0.36	0.014	0.013
Crude ash, % DM	4.80	4.54	4.74	0.08	0.356	0.348

Peer data with different acronyms indicate significant differences (P < 0.05), with different acronyms indicating highly significant differences (P < 0.01) and the same acronym indicating insignificant differences. SEM is the pooled standard error between groups; the P-value indicates significance, and the contrast P-value represents the significance between the control and treatment.

**Table 7:** Effects of different proportions of FPR on the amino acid composition of Simmental bull.

**Project**	**CON**	**Treatment**		**SEM**	***P*-value**	**Contrast *P***
						**CON vs. T**
		**T25**	**T50**			
Alanine, g/100 g	4.27	4.36	4.44	0.04	0.227	0.139
Arginine, g/100 g	4.78	4.89	4.99	0.04	0.162	0.088
Aspartate, g/100 g	6.80	6.79	7.00	0.08	0.446	0.554
Cysteine, g/100 g	0.71^b^	0.81^ab^	0.89^a^	0.03	0.030	0.016
Glutamate, g/100 g	11.26	11.21	11.16	0.22	0.984	0.880
Glycine, g/100 g	3.09	3.21	3.24	0.03	0.125	0.046
Histidine, g/100 g	3.01	3.13	3.18	0.03	0.067	0.027
Isoleucine, g/100 g	3.62	3.60	3.69	0.04	0.620	0.715
Leucine, g/100 g	6.22	6.36	6.47	0.06	0.191	0.102
Lysine, g/100 g	6.89	7.08	7.18	0.07	0.210	0.100
Methionine, g/100 g	2.03	2.07	2.10	0.03	0.545	0.322
Phenylalanine, g/100 g	3.05^b^	3.38^a^	3.24^ab^	0.05	0.016	0.008
Proline, g/100 g	2.9^Bb^	3.04^a^	3.08^Aa^	0.02	0.002	<0.001
Serine, g/100 g	2.69	2.72	2.85	0.03	0.104	0.166
Threonine, g/100 g	3.39	3.45	3.57	0.04	0.130	0.125
Tyrosine, g/100 g	2.58^b^	2.83^a^	2.74^ab^	0.04	0.028	0.012
Valine, g/100 g	3.76	3.84	3.87	0.03	0.387	0.190
EAA, g/100 g	28.96	29.72	30.12	0.27	0.210	0.099
NEAA, g/100 g	42.09	42.76	43.50	0.42	0.417	0.263
DAA, g/100 g	31.06	31.51	31.82	0.34	0.671	0.421
TAA, g/100 g	71.05	72.48	73.62	0.67	0.302	0.167
EAA/NEAA, %	68.81	69.53	69.31	0.39	0.751	0.475
EAA/TAA, %	40.76	41.01	40.92	0.13	0.751	0.482
DAA/TAA, %	43.71	43.46	43.21	0.18	0.548	0.349

Peer data with different acronyms indicate significant differences (P < 0.05), with different acronyms indicating highly significant differences (P < 0.01) and the same acronym indicating insignificant differences. SEM is the pooled standard error between groups; the P-value indicates significance, and the contrast P-value represents the significance between the control and treatment.

### Rumen fermentation parameters

The FPR increases (*P* < 0.05) the rumen fluid pH while CON was 7.05, T25 was 7.18, and T50 was 7.26 (Table 8). The concentrations of isobutyric acid and isovaleric acid significantly (*P* < 0.05) decreased in T25 (0.83 mmol/L) and T50 (0.70 mmol/L) while CON was 0.94, whereas isovaleric acid descent (*P* < 0.05) in T25 (0.88 mmol/L) and T50 (0.62 mmol/L) while CON was 1.92. Butyrate raised (*P* < 0.05) while CON was 7.60%, T25 was 8.87%, and T50 was 9.65%. Thus, FPR had a regulating effect on the fluctuation range of the rumen fermentation parameters.

**Table 8:** Effects of FPR addition on rumen fermentation parameters.

**Project**	**CON**	**Treatment**		**SEM**	***P*-**	**Contrast *P***
					**value**	**CON vs. T**
		**T25**	**T50**			
pH	7.05^b^	7.18^ab^	7.26^a^	0.04	0.042	0.018
NH_3_-N, mg/100 ml	8.81	7.56	6.51	0.52	0.196	0.107
Lactic acid, mg/L	2414.45	2536.24	2360.25	125.16	0.858	0.902
TVFA, mmol/L	46.30	45.66	43.31	2.16	0.850	0.701
Acetic acid, mmol/L	32.94	32.41	30.86	1.51	0.856	0.695
Propionic acid, mmol/L	7.55	7.07	6.73	0.39	0.693	0.435
Isobutyric acid, mmol/L	0.94^a^	0.83^ab^	0.70^b^	0.04	0.038	0.029
Butyrate, mmol/L	3.51	4.11	4.16	0.23	0.434	0.202
Isovaleric acid, mmol/L	1.02^a^	0.88^ab^	0.62^b^	0.06	0.017	0.024
Valeric acid, mmol/L	0.33	0.36	0.25	0.02	0.146	0.534
Acetate to propionate ratio	4.37	4.64	4.68	0.08	0.207	0.081

Peer data with different acronyms indicate significant differences (P < 0.05), with different acronyms indicating highly significant differences (P < 0.01) and the same acronym indicating insignificant differences. SEM is the pooled standard error between groups; the P-value indicates significance, and the contrast P-value represents the significance between the control and treatment.

### 16S rRNA sequencing and annotation analysis

**Figure 2:**
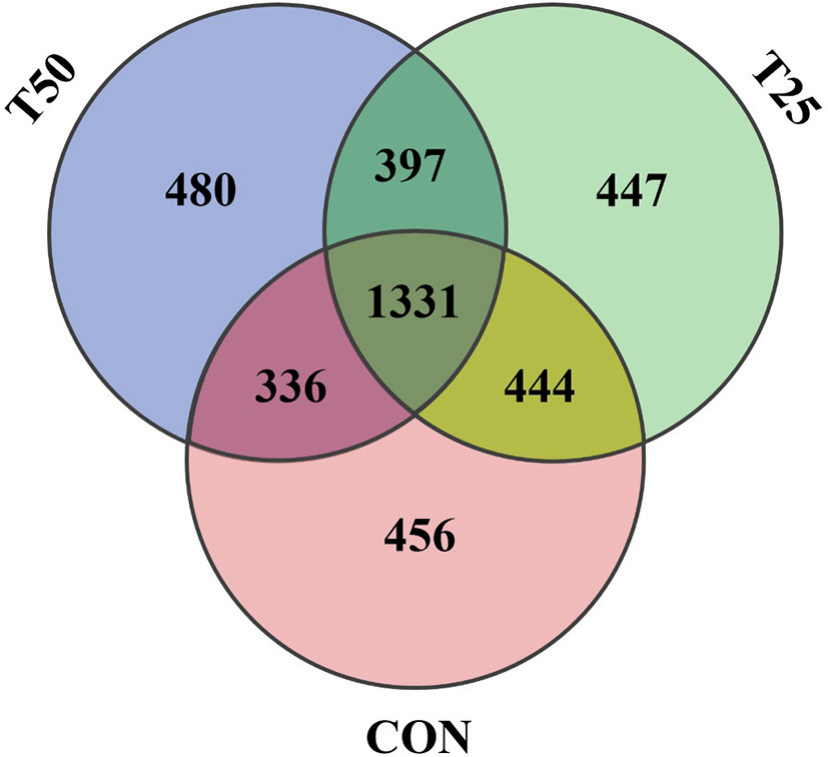
Venn diagram of OTU statistics of rumen bacteria.

**Figure 3:**
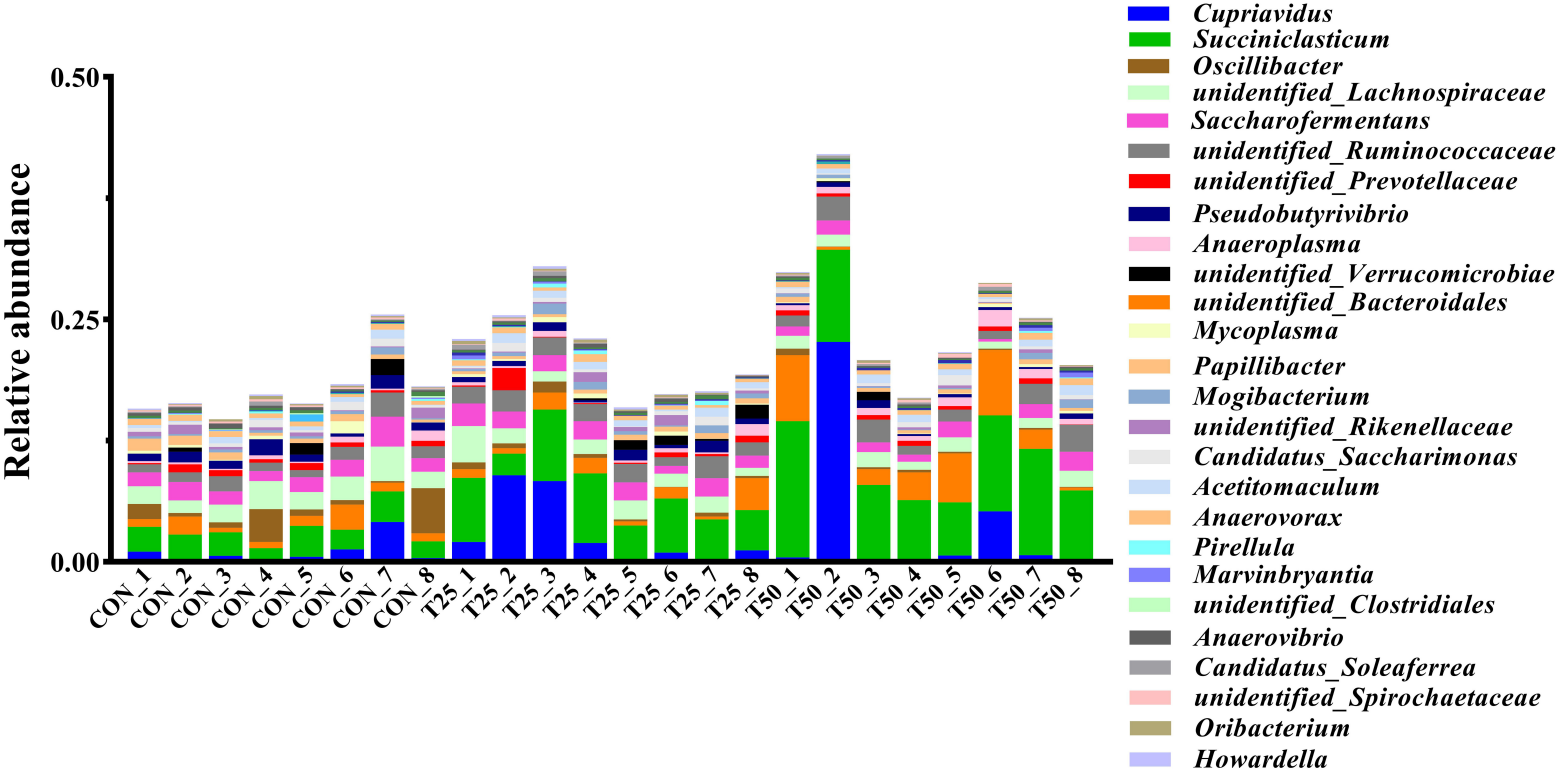
The abundance of the rumen flora in genus level.

**Figure 4:**
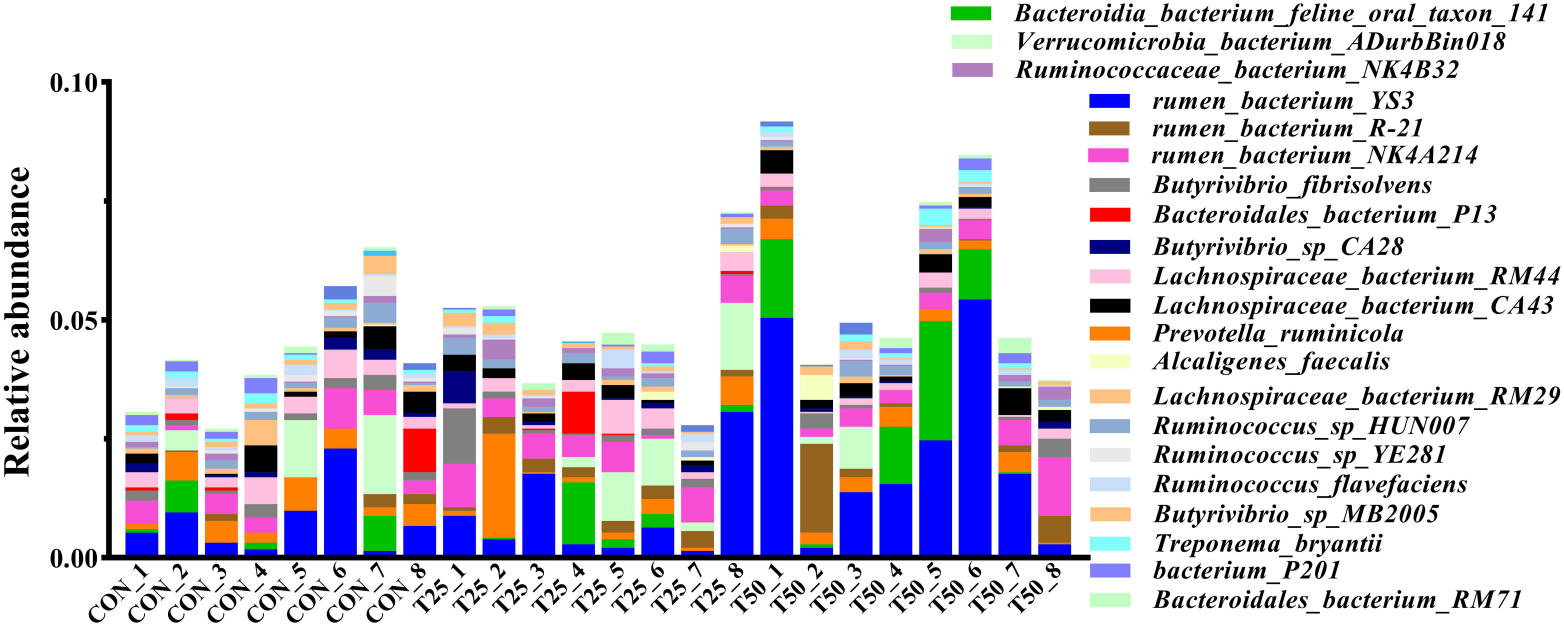
The abundance of the rumen flora in species level.

The V1–V9 regions of the 16S rDNA were enriched, and 356,162 raw reads were collected using high-throughput analysis. After quality control, each sample produced 11,339 valid sequences with a read length of 1,447 nucleotides. Venn diagram analysis yielded 4,119 unique OTU candidates with 97% sequence similarity, and 1,331 candidates shared across all samples were defined as core OTUs (Figure 2). The core OTUs were ~32.31% of the total candidates, whereas 456, 447, and 480 OTUs were identified as unique in the CON, T25, and T50 groups, respectively. A total of 21 phyla, 26 kingdoms, 46 orders, 66 families, 102 genera, and 97 species were found using the OTU annotations. The main bacterial phyla were *Firmicutes, Bacteroidetes*, and *Tenericutes* (48.62, 38.19, and 5.65%, respectively; Figure 3). At the species level (Figure 4), *Rumen_bacterium_YS3* (1.32%) was the most common species. Unclassified bacteria accounted for 93.45% of the OTUs, while the identified secondary strains accounted for 97.03%.

### Microbial diversity in the ruminal fluid of the Simmental bull

**Figure 5:**
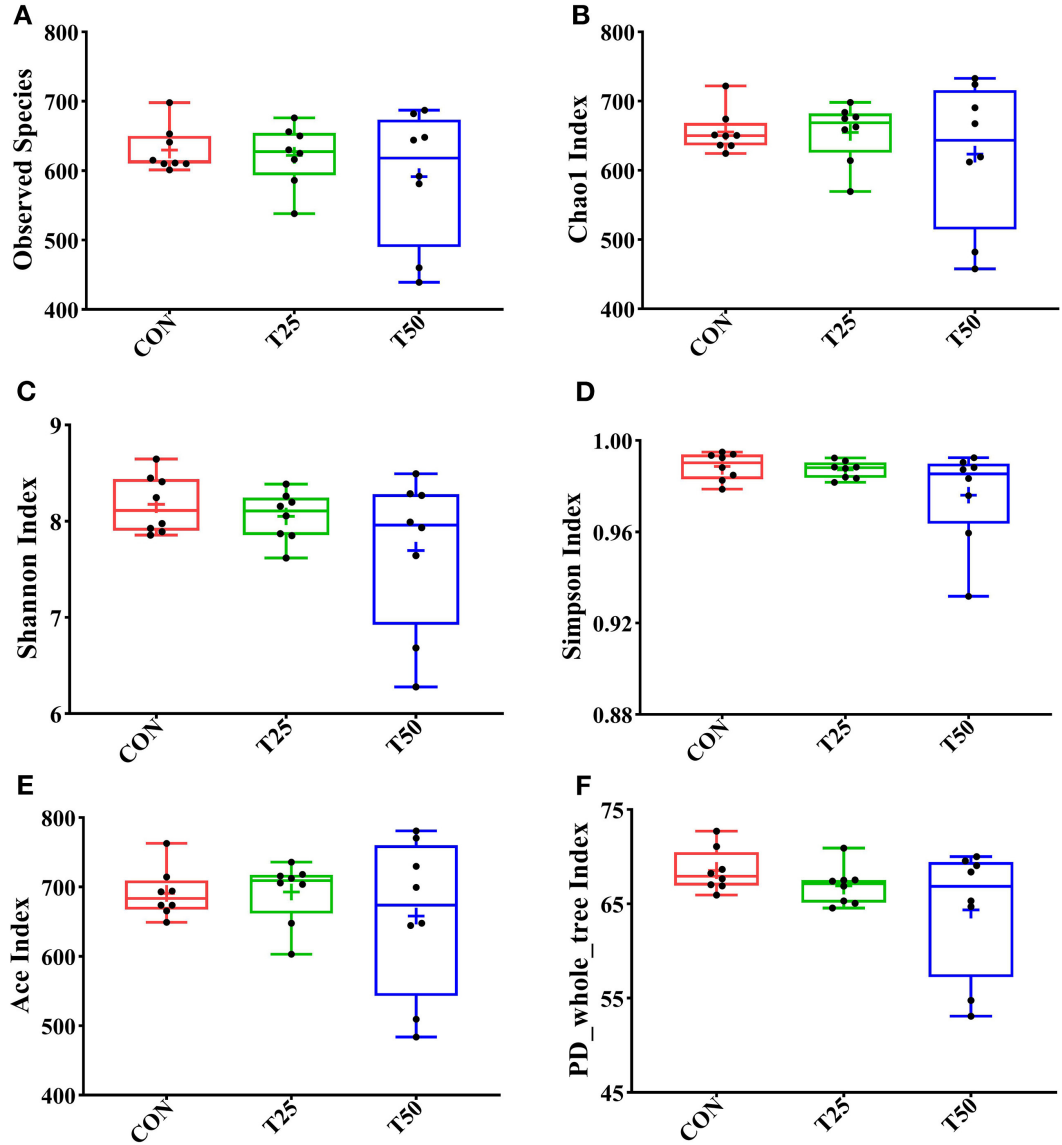
Alpha diversity analysis of rumen flora. **(A)** The number of observed species; **(B)** Chao1 index of species richness; **(C)** Shannon index of species diversity; **(D)** Simpson index of diversity; **(E)** Ace index of species richness; and **(F)** phylogenetic tree index. The “+” symbol in the box plot represents the mean of the within-group exponent.

**Figure 6:**
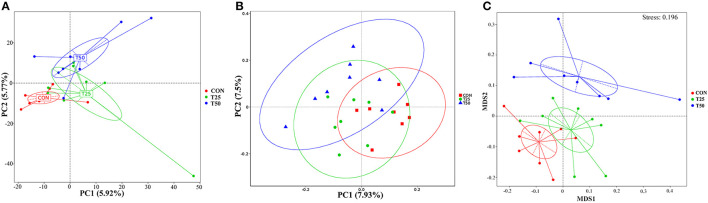
Differences of bacteria between groups. **(A)** PCA main coordinate axis analysis; **(B)** PCoA main coordinate axis analysis; **(C)** NMDS nonmetric multidimensional scale analysis.

Observed species, Chao1, Shannon, Simpson, ACE, and PD_whole_tree were used to evaluate the microbial diversity after FPR treatment (Figure 5). The addition of FPR had no significant effect on the above-mentioned indexes, but the diversity and richness tended to decrease as the proportion of FPR increased. The diversity and richness of the T50 group were the lowest. The rumen flora of the groups was roughly distributed in the same area (Figure 6A). The sample distances were more concentrated within each group, presenting three different colonies as a whole; this indicated that FPR affected the main bacterial groups in the rumen. The locations of the sample points in each group were not completely separated (Figure 6B), and the area of intersection of sample colonies in each group was the smallest for CON and T50, which indicated that 25% FPR affected more than 50% but not vigorously. The differences between and within bacterial groups showed obvious discrimination under the nonlinear structure (Figure 6C); the samples were clustered more centrally within each treatment, and the groups were well distinguished.

**Figure 7:**
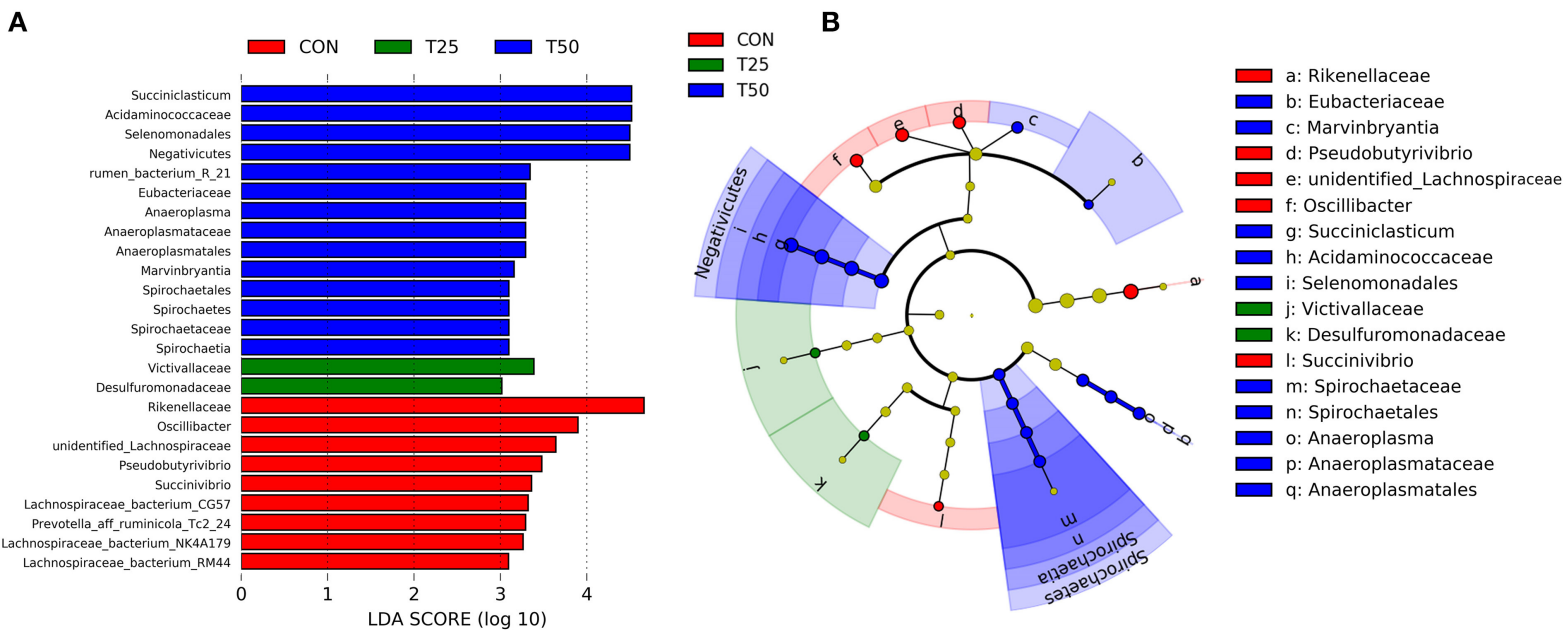
LEfSe analysis of rumen microflora. **(A)** LDA bar chart; **(B)** LEfSe evolution branch diagram. The graph reflects the affiliation of flora populations between groups at the species to phyla level, with node size corresponding to the average relative abundance of the corresponding taxon.

A total of 25 different bacterial strains were statistically detected between the groups, with nine species in CON, two in T25, and 14 in T50 (Figure 7A). The phylogenetic tree showed multiple clades (Figure 7B). The evolutionary routes of the three treatment groups were mixed with each other, indicating they had similar evolutionary directions. This showed that the environment created in the rumen was not the same when the proportion of FPR was different, so the rumen microbes followed different evolutionary directions.

### Correlations between rumen microbiota and rumen fermentation parameters

**Figure 8:**
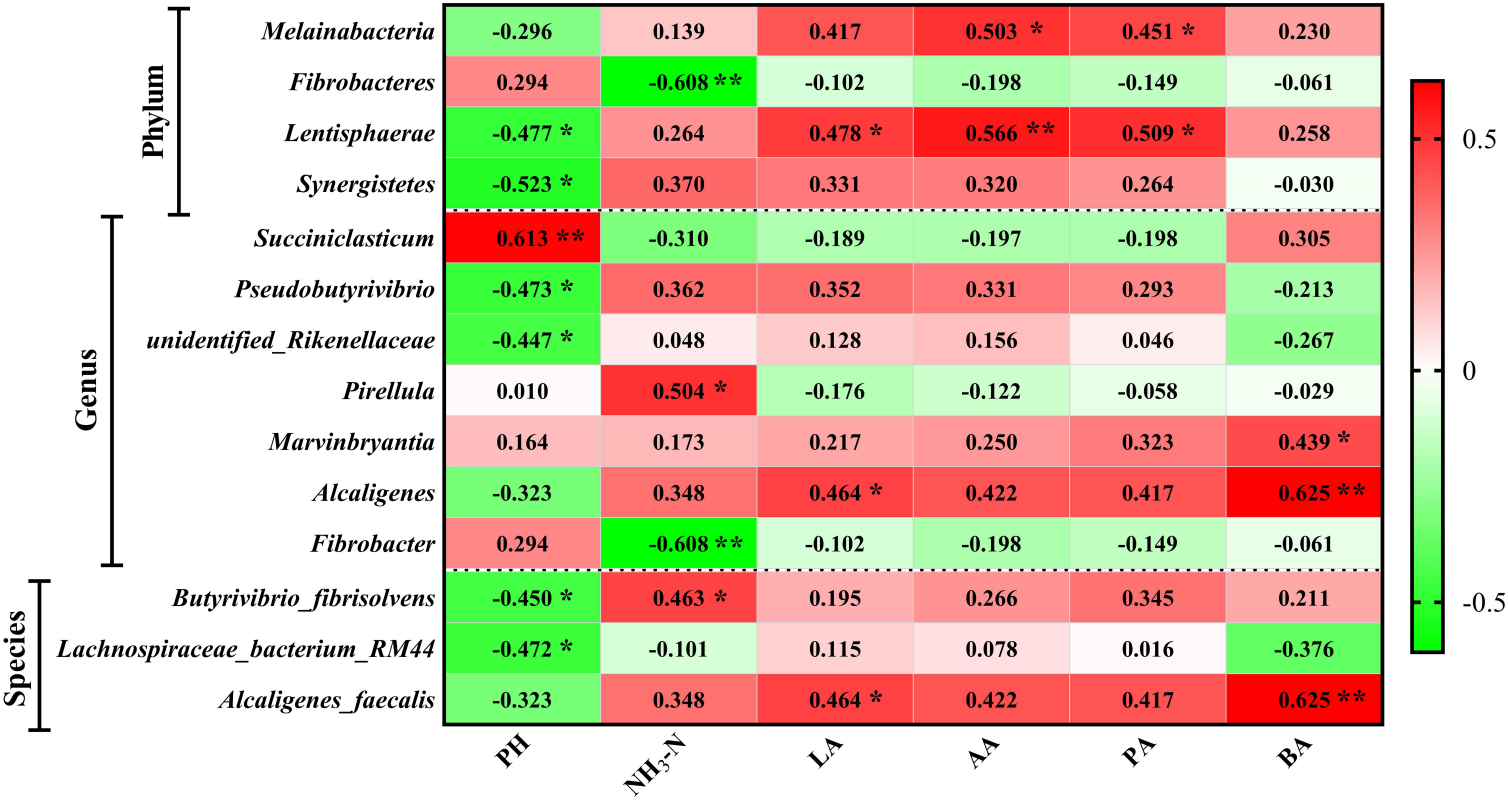
Correlation of fermentation parameters and flora. *indicates *P* < 0.05, and **indicates *P* < 0.01.

To analyze the correlations of FPR and rumen microbiota, Pearson correlation analysis was performed and then found that four phyla, seven genera, and three species were related to the rumen fermentation parameters (Figure 8). At the genus level, *unidentified_Rikenellaceae* and *Psedobutyrivibrio* were positively (*P* < 0.05) correlated to pH, whereas *Succiniclasticum* was significantly (*P* < 0.05) negatively correlated. *Fibrobacter* was negatively (*P* < 0.05) correlated to NH3-N while *Pirellula* was positive. *Alcaligenes* was positively (*P* < 0.05) correlated to lactic acid and butyric acid. *Marvinbryantia* was correlated to butyric acid positively (*P* < 0.05). At the species level, *Lachnospiraceae_bacterium_RM* and *Butyrivibrio_fibrisolvens* were negatively (*P* < 0.05) correlated to pH. *Butyrivibrio_fibrisolvens* was positively (*P* < 0.05) correlated to NH_3_-N. *Alcaligenes_faecalis* was significantly correlated to lactic acid and butyric acid (*P* < 0.05).

## Discussion

Agricultural by-products may be used to solve the feed shortage problem. In this study, the nutrient levels of FPR (~21% DM, 6% CP, 63% NDF, and 33% ADF) were lower than SPR (~32% DM, 7% CP, 72% NDF, and 43% ADF). However, the addition of FPR resulted in higher average daily weight gain and a lower DMI-weight-gain ratio. Thus, the treatments generated more net profit. This could be attributed to the addition of lactic acid bacteria and yeast to the pineapple residue during fermentation, which may have improved the digestibility of feed and energy efficiency in rumen (Niba et al., 2009; Kaewpila et al., 2021; So et al., 2021), cause the organic acids produced during fermentation contributed on structural carbohydrate hydrolysis (Wang et al., 2018). The addition of FPR had no adverse effects on the slaughter performance and beef sensory quality, which is similar to the findings of Hattakum et al. (2019), Liu et al. (2020), and Mello et al. (2021). Intramuscular fat deposition is influenced by numerous factors, such as breed, genotype, age, and nutrition (Jeong et al., 2013). Luccia et al. (2013) and Zhang and Guan (2019) found that improving dietary energy levels would increase the intramuscular fat content and decrease shear force, thus improving meat quality. A significant increase in crude fat content was observed in T50, whereas no differential shear force was detected. The possible mechanism of this strange phenomenon needs further study.

Isovalerate and isobutyrate are branched-chain VFAs (BCVFAs) produced by rumen microbial deamination and decarboxylation of leucine and valine. BCVFAs can improve NDF degradability, bacterial protein synthesis, and bacterial growth rate (Kajikawa et al., 2002; Zhang et al., 2013). Liu et al. (2020) found the addition of BCVFAs accelerated the growth of Holstein dairy calves. However, this study showed that isobutyric acid and isovaleric acid decreased significantly in the treatments. Considering the higher feed conversion rate in treatments, the relevant BCVFAs dynamically change in a complex process when the NDF reaches a lower level. The OTUs of the groups were relatively similar, but the components of the microflora were significantly different, with T50 showing the lowest diversity and richness. This was also reflected in the changes in the rumen microecological environment, such as pH (CON, 7.05; T25, 7.18; T50, 7.26) and NH_3_-N (CON, 8.81; T25, 7.56; T50, 6.51; mg/100 ml). Therefore, the phylogenetic composition of the rumen microbes was quite different.

Members of *Succiniclasticum* are involved in converting succinate to propionate and contributing to fiber metabolism in ruminants (van Gylswyk et al., 1997). Furthermore, *Succiniclasticum* abundance has been positively correlated to feed efficiency (Auffret et al., 2020; Clemmons et al., 2020). Daghio et al. (2021) and Du et al. (2021) found that *Succiniclasticum* was positively correlated to body weight, which is consistent with our results. Ma et al. (2021) observed that *Succiniclasticum* was positively related to NH_3_-N, isobutyrate, and isovalerate levels, which is inconsistent with our results. *Lachnospiraceae* has been reported to be correlated to feed efficiency in beef cattle (Li and Guan, 2017; Hernandez et al., 2021). The decreased relative abundance of *unidentified_Lachnospiraceae* shows that FPR contributes to intestinal health. Ma et al. (2019) found that *unidentified_Lachnospiraceae* was positively correlated to SOD and GSH when mice were fed with a high-fat diet, but it was negative in this study. It concluded that the FPR contributes to reducing oxidative stress damage in the gut. The increased relative abundance of *Oscillibacter* may lead to metabolic diseases (Naseribafrouei et al., 2014; Cheung et al., 2019). *Pseudobutyrivibrio* is related to sugar phosphorylation metabolism (Kasperowiczb et al., 2010), and *Anaeroplasma* has been found to be associated with lipid metabolic diseases and short-chain fatty acid metabolism (Granado-Serrano et al., 2019; Velazquez et al., 2019). This may partly explain the decline of isobutyric acid and isovaleric acid varied in T50 cause.

## Conclusion

The results indicate that FPR increased growth performance and did not affect the major VFA content of the rumen or the diversity and richness of the rumen flora. The net profit of each bull in treatments had improved. Synthetically considering the economic benefits and growth performance, 25% FPR in diet has a positive impact on feeding bull. However, the specific mechanisms need to be studied further.

## Data availability statement

The original contributions presented in the study are included in the article/supplementary material, further inquiries can be directed to the corresponding authors.

## Ethics statement

The animal study was reviewed and approved by Administration of Laboratory Animals (CLI.2.293192, 2017 Revision, State Council, China) Institutional Animal Care and Use Committees of South China Agricultural University (Approval No. 2018-P002).

## Author contributions

This study was conceived and designed by YL and DL. The experiments was performed by ZY and MD. The data were analyzed by ZY and ZX. The manuscript was mainly written by ZY and ZX with the assistance of GL, BS, YG, and YL. All authors read and approved the final manuscript.

## Funding

This study was supported by the Guangdong Provincial Promotion Project of Modern Seed Industry, GuangDong Basic and Applied Basic Research Foundation-Enterprise (Wens) Joint Fund (2019B1515210017), Special Fund of Agricultural Development and Rural Work in Guangdong Province Beef Cattle Concentrate Feed and Roughage Research and Development, and Popularization and Application in South China.

## Conflict of interest

The authors declare that the research was conducted in the absence of any commercial or financial relationships that could be construed as a potential conflict of interest.

## Publisher's note

All claims expressed in this article are solely those of the authors and do not necessarily represent those of their affiliated organizations, or those of the publisher, the editors and the reviewers. Any product that may be evaluated in this article, or claim that may be made by its manufacturer, is not guaranteed or endorsed by the publisher.

## Abbreviations

HSPD1: heat shock protein family D member 1
SIFs: sperm intrinsic factors
FPR: fermented pineapple residue
SPR: silage *Pennisetum sinese* Roxb
DM: dry matter
CP: crude protein
EE: ether extract
NDF: neutral detergent fiber
ADF: acid detergent fiber
VFA: volatile fatty acid
NH_3_-N: ammonia nitrogen
OTU: operational taxonomic unit
DMI: dry matter intake.
